# IL-6/STAT3/TWIST inhibition reverses ionizing radiation-induced EMT and radioresistance in esophageal squamous carcinoma

**DOI:** 10.18632/oncotarget.14495

**Published:** 2017-01-04

**Authors:** Chunbao Zang, Xujie Liu, Bing Li, Yanqiong He, Shen Jing, Yujia He, Wenli Wu, Bingqian Zhang, Shuhong Ma, Weiwei Dai, Shaolin Li, Zhiping Peng

**Affiliations:** ^1^ Department of Radiological Medicine, Chongqing Medical University, Chonging, China; ^2^ Department of Otorhinolaryngology, The First Affiliated Hospital of Chonqqing Medical University, Chongqing, China

**Keywords:** radioresistance, EMT, IL-6/STAT3/TWIST pathway

## Abstract

The acquisition of radioresistance by esophageal squamous carcinoma (ESC) cells during radiotherapy may lead to cancer recurrence and poor survival. Previous studies have demonstrated that ionizing radiation (IR) induces epithelial–mesenchymal transition (EMT) of ESC cells accompanied by increased migration, invasion, and radioresistance. However, the underlying molecular mechanisms of IR-induced EMT and radioresistance are not well established, hampering the development of potential solutions. To address this issue, we investigated the role of the IL-6/STAT3/TWIST signaling pathway in IR-induced EMT. We found not only the pathway was activated during IR-induced EMT but also STAT3 inhibition or Twist depletion reversed the EMT process and attenuated radioresistance. These results improve our understanding of the underlying mechanisms involved in IR-induced EMT and suggest potential interventions to prevent EMT-induced acquisition of radioresistance.

## INTRODUCTION

Esophageal squamous carcinoma (ESC) is one of the most common malignancies worldwide and has a very high morbidity and mortality rate [1]. In northern China, 250,000 new cases are diagnosed per year, accounting for more than half of the global incidence [2]. In recent years, more accurate intensity-modulated radiation therapy combined with adjuvant chemotherapy has improved the ESC cure rate; however, the 5-year survival rate is still less than 30% due to acquired radioresistance and chemoresistance [3]. Therefore, overcoming therapy resistance is one of the key solutions to conquering cancer.

Epithelial–mesenchymal transition (EMT) is a crucial cellular process in which cells change their original epithelial phenotype and adopt the characteristics and morphology of mesenchymal cells [4, 5]. The transition is accompanied by decreased expression of epithelial-specific biomarkers, such as E-cadherin, β-catenin, and occludin, and increased expression of mesenchymal-specific biomarkers, such as N-cadherin, vimentin, fibronectin, and TWIST [6]. Recently, numerous studies have demonstrated that exposure to ionizing radiation (IR) can induce human cancer cells to undergo EMT, which leads to acquired radioresistance [7–10]. Therefore, it is necessary to explore the potential mechanisms of IR-induced EMT and to search for possible solutions to reverse the resulting radioresistance.

A number of well-studied cytokines have been implicated in the EMT process in carcinoma cell lines [4, 11–15]. One of these, IL-6, is a pleiotropic cytokine that enhances the proliferation of cancer cells [16] and, reportedly, plays a central role in EMT in breast cancer [17]. IL-6 signaling occurs through a specific cell surface receptor, IL-6R (CD126), coupled to a transmembrane signal transducer, gp130 (CD130) [18, 19], which activates the JAK/STAT3 signaling pathway. Previous studies have shown that sustained IL-6 expression can constitutively activate STAT3 [12, 17, 20], which transactivates Twist gene expression and promotes carcinoma cell migration and invasion, as well as resistance to drug and radiation therapy [21].

Taking these studies into consideration, we hypothesized that IR might induce EMT and subsequent radioresistance in ESC cells by activating the IL-6/STAT3/TWIST signal pathway. In this study, we confirmed that ESC cells undergo EMT and acquire radioresistance after exposure to IR. We also demonstrated that IL-6/STAT3/TWIST activation is the key event involved in IR-induced EMT and radioresistance, and that inhibition of activated STAT3 or depletion of TWIST prevented the transition. Taken together, our findings uncover an important mechanism for the acquisition of radioresistance and suggest that inhibition of the IL-6/STAT3/TWIST pathway may be a promising novel intervention to prevent radioresistance.

## RESULTS

### EMT phenotypes and gene expression induced by IR

**Figure 1:**
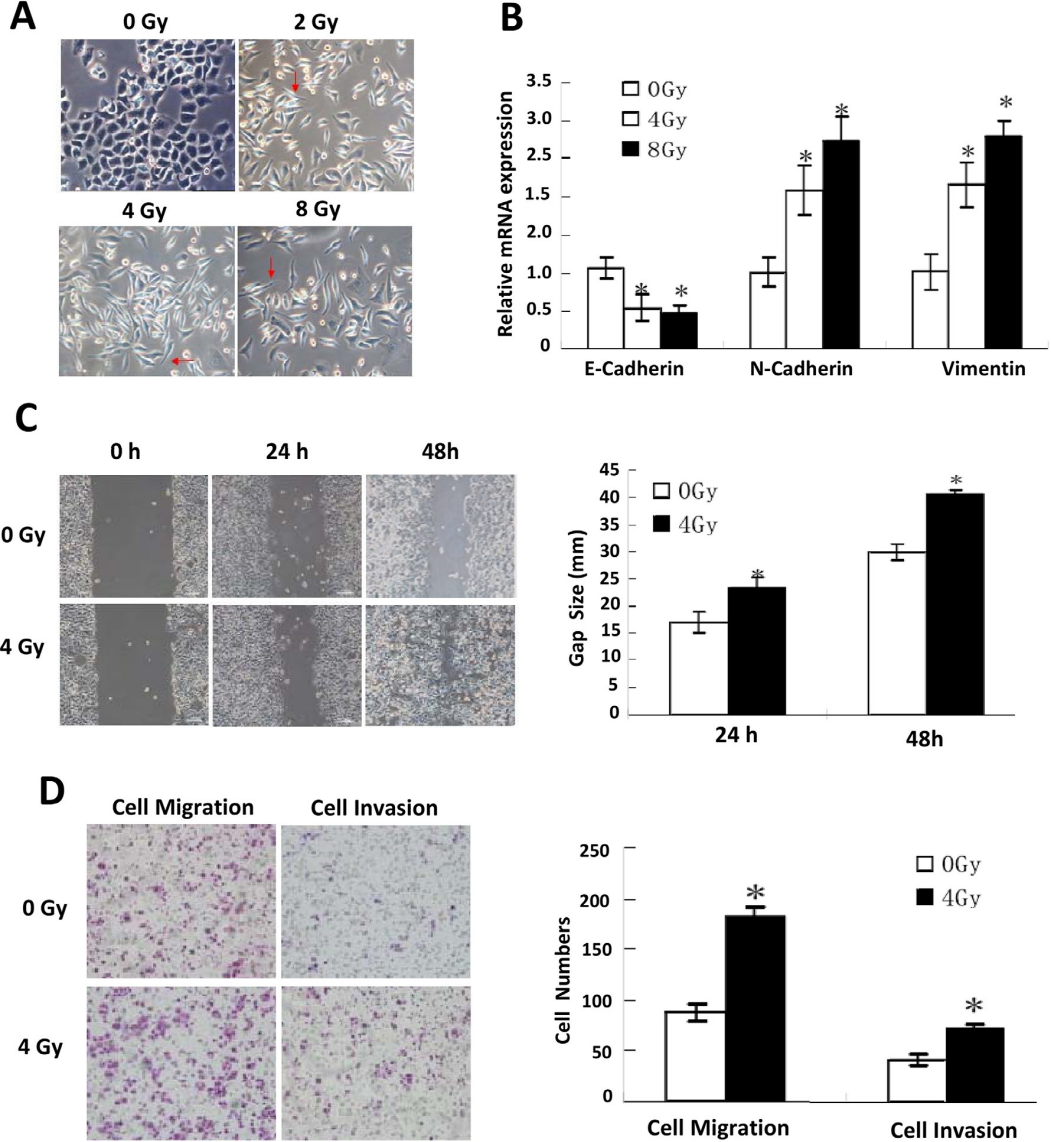
EMT phenotypes displaying **(A)** Representative morphological changes in IR-treated Eca109 cells. **(B)** Relative mRNA expression of the EMT biomarkers: E-cadherin, N-cadherin and vimentin detected by quantitative PCR under different doses of IR treatment. **(C)** Scratch assay detection of cells’ invasiveness under different doses of IR treatment (left), and their quantitative analysis (right). **(D)** Transwell invasion assay detection of cells’ migration and invasion ability (left), and their quantitative analysis (right).

As shown in Figure 1A, Eca109 cells under 0 Gy were oval with tight cell-to-cell junctions, whereas the morphology of 2, 4, and 8 Gy IR-treated cells was markedly different. These cells exhibited typical ‘cobblestone’ morphology with a long, narrow, and spindle-like shape, extended pseudopodia, and dilated intercellular spaces. These results are consistent with the classic morphological changes associated with EMT. Cells received increasing irradiation doses (2, 4 and 8 Gy) generally presented similar morphology changes. The subtle differences between them were that cells showed longer and narrower shape along with increased irradiation doses.

To investigate whether gene expression in IR-treated cells and tumors from ESC patients after radiotherapy was consistent with EMT, we examined epithelial and mesenchymal biomarker expression using a panel of tests. The results of the Q-PCR (Figure 1B), IFA, and IHC assays and western blotting (Supplementary Figure 1A, 1B, 1C, 1D) showed that E-cadherin expression was reduced, whereas N-cadherin and vimentin expression were enhanced, confirming EMT was induced by IR.

### Enhanced invasiveness and activated IL-6/STAT3/TWIST pathway

Experiments were performed at 24 or 48 h after the end of IR. The invasiveness and metastatic capability of the cells was evaluated by scratch assay and Transwell invasion assay. As shown in Figure 1C, the scratch assay showed markedly enhanced migration in IR-treated cells, and similar results were obtained in the Transwell invasion assay (Figure 1D).

**Figure 2:**
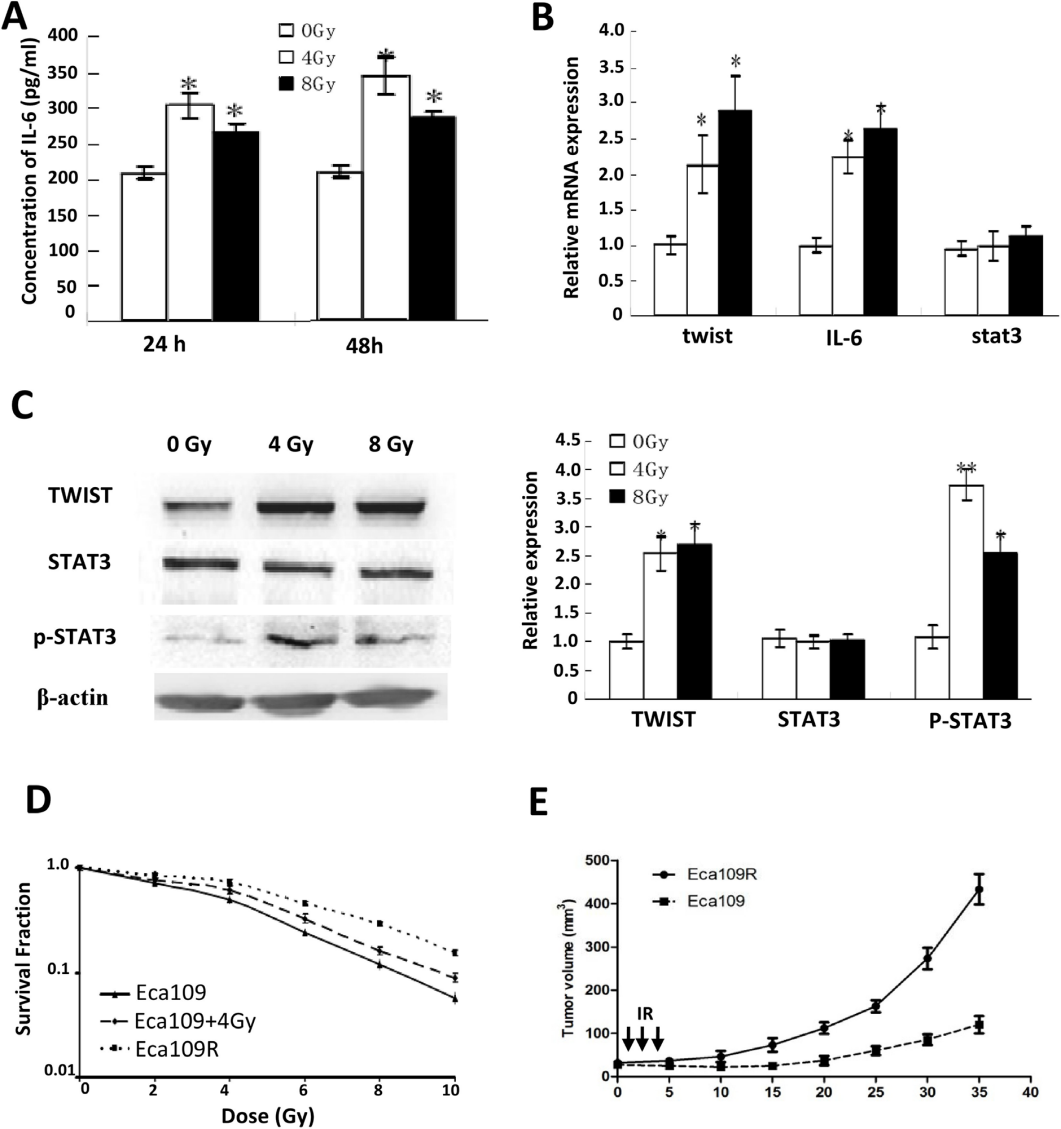
Activated IL-6 Pathway and acquired resistance evaluation **(A)** IL-6 concentrations detected by ELISA. **(B)** Quantitative PCR detection of twist, IL-6 and stat3 mRNA. **(C)** Western-blotting detection of TWIST, STAT3 and p-STAT3 expression (left), and their quantitative analysis (right). **(D)** Surviving fraction and radiobiology parameters of cells detected by colony-forming assay. **(E)** Growth curves of parental Eca109 and Eca109R xenografted tumors.

IL-6 concentrations in the culture medium were markedly increased, as detected by ELISA (Figure 2A and Supplementary Table 2), suggesting that the IL-6 signaling pathway might be activated. Consistent with this, expression of the downstream effectors p-STAT3 (705) and TWIST was significantly enhanced, as detected by Q-PCR, western blotting (Figure 2B, 2C) and IHC for tumors from ESC patients after radiotherapy (Supplementary Figure 1E).

### Radioresistance acquisition accompanied by EMT displaying

Colony-forming assay was performed to confirm Eca109R have obtained radioresistance, the results showed the surviving fraction and radiobiology parameters (SF2,D0,Dq, and N) of Eca109R and Eca109 cells exposed to 4 Gy IR (Eca109 + 4 Gy) were significantly increased compared with untreated Eca109 cells, while the radiosensitivity enhancement ratios (SERD0 and SERDq) were decreased (Figure 2D, Supplementary Table 3). The *in vivo* experiment for tumors growth curve of Eca109 and Eca109R xenografts confirmed the acquired radioresistance (Figure 2E).

**Figure 3:**
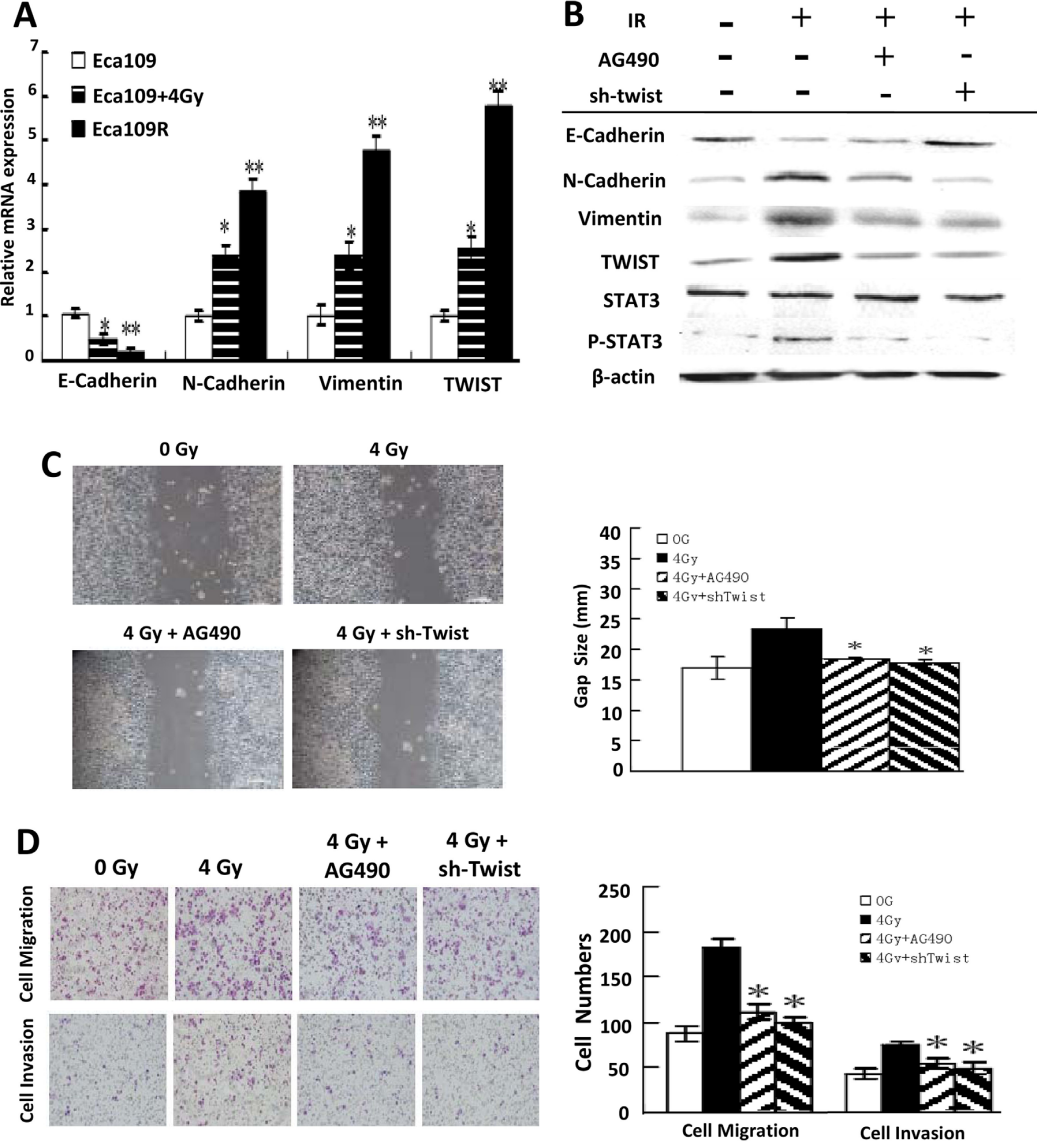
EMT reversion by IL-6 Pathway Blockade **(A)** Quantitative PCR detection of the EMT biomarkers mRNA in each cell line. **(B)** Western-blotting detection of IL-6 pathway inhibition and EMT biomarkers expression change in Eca109 cells exposed to IR, AG490 or sh-Twist. **(C)** Scratch assay detection of attenuated invasiveness of EMT-Eca109 cells under AG490 or sh-Twist treatment (left) and their quantitative analysis (right). **(D)** Transwell invasion assay detection of impaired migration and invasion ability of EMT-Eca109 cells under AG490 or sh-Twist treatment (left) and their quantitative analysis (right).

Furthermore, along with radioresistance acquiring, both Eca109R and Eca109 cells that were exposed to 4 Gy IR displayed EMT phenotype (Figure 3A, Supplementary Figure 2A, 2B), which are similar to previous results, suggesting that radioresistant cells underwent EMT.

### Blockade of the IL-6 pathway reverses EMT

To investigate whether IR-induced EMT can be reversed by blocking IL-6/STAT3/TWIST pathway, cells were incubated with the Jak2 inhibitor AG490 to block STAT3 activation or transfected with a plasmid encoding twist-specific shRNA to inhibit TWIST expression. Actually, all four shRNA tested were shown to be effective in reducing twist mRNA expression, as measured by Q-PCR and Western-blotting (Supplementary Figure 2C, 2D). sh-Twist-996, the most efficient of the four, was chosen for following research.

In agreement with our expectations, blockade of the IL-6 pathway reversed IR-induced EMT. Thus, IL-6/STAT3/TWIST pathway blockade was accompanied by induction of E-cadherin and repression of N-cadherin and vimentin both in cells (Figure 3B) and xenografted tumors (Supplementary Figure 3A) and impaired invasiveness and metastatic ability (Figure 3C, 3D).

**Figure 4:**
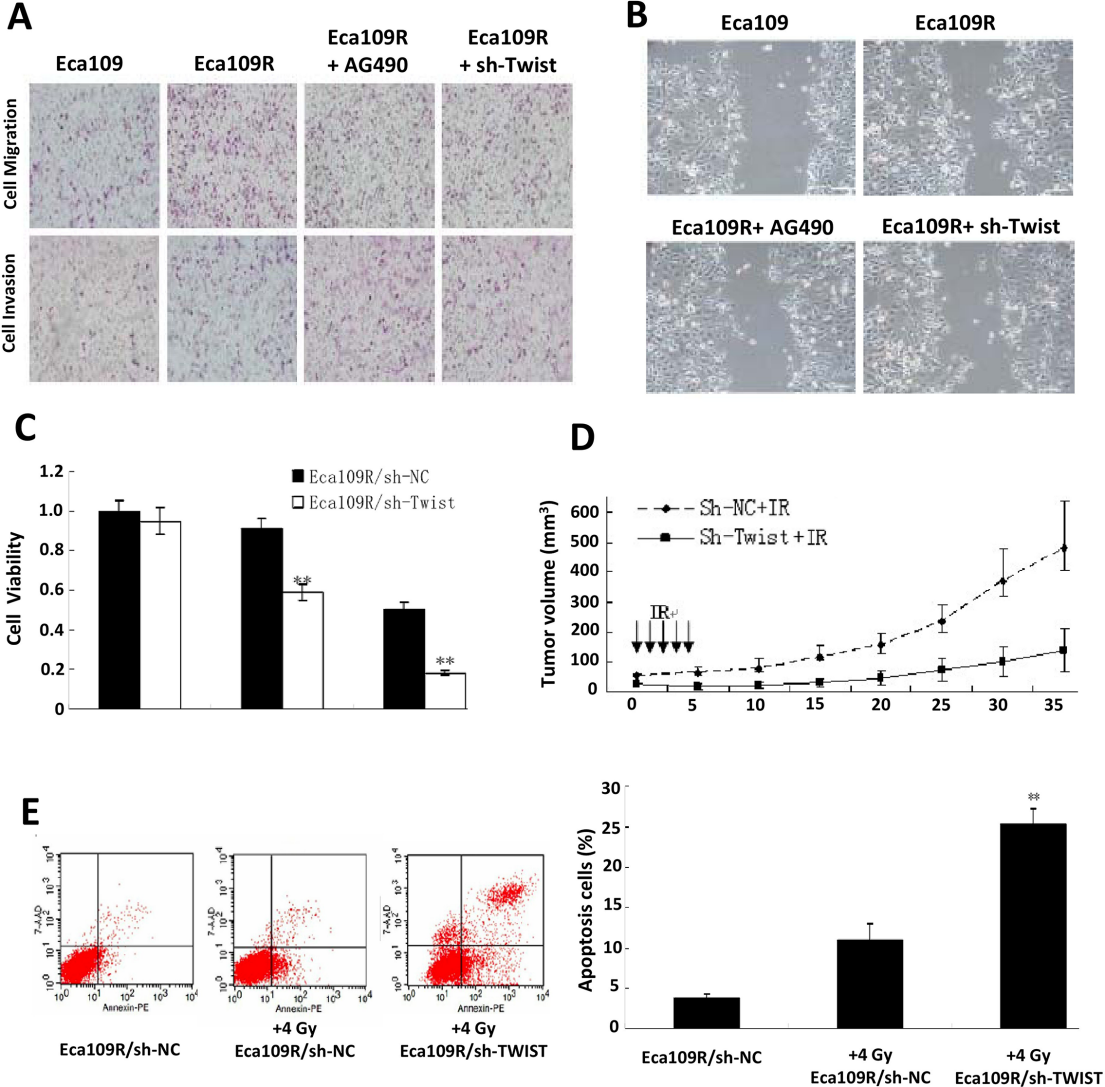
Acquired radioresistance reversion by IL-6 Pathway inhibition **(A)** Representative impaired migration and invasion ability of AG490 or sh-Twist treated Eca109R cells. **(B)** Representative attenuated invasiveness images of AG490 or sh-Twist treated Eca109R cells. **(C)** Attenuated cell viability of Eca109R under sh-Twist transfection detected by MTT assay. **(D)** In vivo IR treatment analysis evaluated radioresistance reversion by sh-Twist. **(E)** FACS analysis of annexin V-stained cells (left), and their quantitative analysis (right).

More importantly, Similar results were also obtained from the Eca109R cells (Figure 4A, 4B and Supplementary Figure 3B), demonstrating that acquired EMT of radioresistant cells could also be reversed by IL-6 pathway inhibition.

### Inhibition of the IL-6 pathway reverses acquired radioresistance

To investigate whether IL-6 pathway inhibition can reverse acquired radioresistance as this pathway was proved to be involved in EMT development according to above results. In line with our expectations, concomitant with EMT reversion, irradiation was sensitized by the pathway inhibition. Thus, colony-forming assays showed a significant decrease in the surviving fraction of Eca109R/sh-Twist cells compared with Eca109R/sh-NC cells (Supplementary Figure 3C, Supplementary Table 5) and the MTT viability assay revealed similar results (Figure 4C). Finally, the increase in radiosensitivity was confirmed *in vivo* by analysis of xenografted tumors exposed to IR. At day 35 post-radiation, the average size of tumors in the Eca109R/sh-Twist + IR mouse group was 140.19 ± 73.25 mm^3^, which was significantly smaller than the average tumor size of 477.89 ± 156.41 mm^3^ in the Eca109R/sh-NC + IR group (Figure 4D).

### Twist silencing promotes apoptosis and inhibits Akt activation

shRNA-mediated silencing of twist was found to promote ESC cell apoptosis, as shown by FACS analysis of annexin V-stained cells (Figure 4E). In addition, western blotting analysis showed that p-AKT and BCL-2 expression was reduced, while BAX and cleaved caspase3 expression was increased, in Eca109R/sh-Twist cells compared with either Eca109R/sh-NC or Eca109 cells after short-term (24 h) exposure to 4 Gy IR (Supplementary Figure 3D). These results suggest that the reversion of IR-induced EMT and radioresistance following twist silencing could be partly attributed to promotion of apoptosis and suppression of proliferation.

## DISCUSSION

Currently, radioresistance remains a major obstacle to the successful treatment of a large number of ESC patients [23, 24], emphasizing the importance and urgency of finding novel methods to enhance the radiosensitivity of ESC. Traditionally, cancer radioresistance can be divided into intrinsic radioresistance and acquired radioresistance. Although the mechanisms of intrinsic and acquired resistance to chemotherapy or radiotherapy have been studied since 1960s [25], the resistance is still being a major impediment in medical oncology. As for intrinsic resistance, two known models, cancer stem cells (CSC) models and the environment-mediated drug resistance (EMDR) model maybe give reasonable explanation to origin of resistant cells. In the CSC model, rare populations of cancer stem cells present multiple chemo-/radio-therapy resistance characteristics: changed cell cycles, over-expressed drug transporters or anti-apoptotic molecules [26–28]. While in the EMDR model, cancer cells interact with the surrounding microenvironment and then enter a quiescent or dormant state as a means of circumventing the effects of the given therapy [29, 30]. While the acquired radioresistance that cancer cells showed sublethal damage repair and accelerated proliferation after IR, was firstly found in the early 1950s [31]. Though more than half century passed, the exact mechanisms of radioresistance acquisition are still unclarified. Numerous studies had been performed to demonstrate its possibility mechanisms in various aspects. Kim et al. [32] found aldehyde reductase expression increased under IR treatment, and then aldehyde reductase inhibited p53 activation finally leading to radioresistance acquisition in laryngeal cancer. Whereas Shimura's study [33] indicated that IR could activate AKT/GSK3β/cyclin D1 pathway resulting in cancer cell enhanced proliferation and radioresistance.

Recent studies suggest that EMT plays a central role in cancer radioresistance development [6, 15, 34]. Increasing evidences have shown that IR contributes to EMT-associated phenotypes, i.e. morphological changes and expression regulation of specific biomarkers [35]. Furthermore, IR-induced EMT has been shown to contribute to the more malignant characteristics of radiation-treated tumors, including enhanced invasiveness, migration, chemoresistance, and radioresistance, which collectively result in tumor metastasis, recurrence, and treatment failure [7, 8, 13, 24, 36, 37]. Thus, EMT seems to be the key process for emergence of radioresistance during radiotherapy and, therefore, we hypothesized that inhibition of EMT could be an attractive strategy for reversing radioresistance.

Sullivan and colleagues [12] first demonstrated in human breast cancer that IL-6 could induce EMT. In their studies, constitutive IL-6 expression activated STAT3 and consequentially promoted and maintained the EMT phenotype. They also found that MCF7 cells, which have aberrant IL-6 production and STAT3 activation, constitutively express TWIST, a direct transcriptional repressor of E-cadherin. Elevated TWIST protein levels are usually associated with cancer cell metastasis and the EMT process. Cheng et al. [21] found that TWIST promotes AKT signaling through transcriptional upregulation of AKT2, and they also showed that IL-6-activated STAT3 could induce TWIST expression. Thus, STAT3, TWIST and AKT form a functional signaling axis that regulates pivotal oncogenic properties of cancer cells. In the present study, we first confirmed that IR exposure induced EMT, elevated IL-6 expression, and activated the IL-6/STAT3/TWIST pathway. Using colony-forming and viability assays, we also proved that cells undergoing IR-induced EMT acquired radioresistance. All these findings led and assure us to investigate the possibility that inhibition of IL-6/STAT3/TWIST signaling might reverse the IR-induced EMT and EMT-associated radioresistance sequentially. In agreement with our expectations, activated STAT3 inhibition by small molecule AG490 or TWIST expression down-regulation by sh-RNA indeed upregulated epithelial specific biomarkers, downregulated mesenchymal specific biomarkers, and furthermore attenuated invasiveness and metastatic ability, strongly suggesting that blockade of this pathway is an effective means to reverse EMT.

To further confirm our hypothesis that EMT reversion leading to radioresistance reversion, we investigated the radioresistant cancer cells’ sensitivity to irradiation under the situation of EMT reversion by IL-6 pathway inhibition. From the results of colony-forming, viability assays and *in vivo* IR treated xenografts analysis, the impairment of radioresistance was observed upon silencing twist expression. Furthermore, we demonstrated that this radioresistance reversion could at least partly be ascribed to inhibition of Akt and promotion of apoptosis. Numerous studies found STAT3 activation participates in regulating expression of apoptosis genes [38]. STAT3 inhibition can even directly induce apoptosis in prostate cancer lines [39]; while persistent activation of STAT3 signaling would confer resistance to apoptosis in human breast cancer cells [40]. In present study, through attenuating TWIST expression by sh-RNA, not only has IL-6/STAT3 signaling pathway been inhibited, but also apoptosis related proteins were activated, which are consistence with previous above-mentioned reports. The exact mechanisms involved IL-6/STAT3/TWIST regulating apoptosis deserves further and deeper study in future.

In present study, we also confirmed irradiation treatment has potential to enhance cancer cell invasion and metastasis again. As a matter of fact, a large number of studies have found sub-lethal doses irradiation could promote different kinds of cancer cells invasive and metastatic [41–44]. Qian et al. [43] confirmed fractionated irradiation enhanced pancreatic cancer cells invasiveness associated with increased expression/activity of MMP-2. Wild-Bode et al. [45] showed that sub-lethal doses of irradiation enhanced human glioblastoma cells migration and invasiveness ascribed to enhanced expression or activity of MMP-2 and MMP-9. Furthermore, Wang et al. [41] research found SDF-1-regulated macrophage mobilization and vasculogenesis involved in irradiation therapy induced tumor invasiveness. All these findings support our present results in esophageal squamous carcinoma cells, while as well imply that more reasonable irradiation dose and therapeutic approaches of the current radiotherapy needs to be improved.

**Figure 5:**
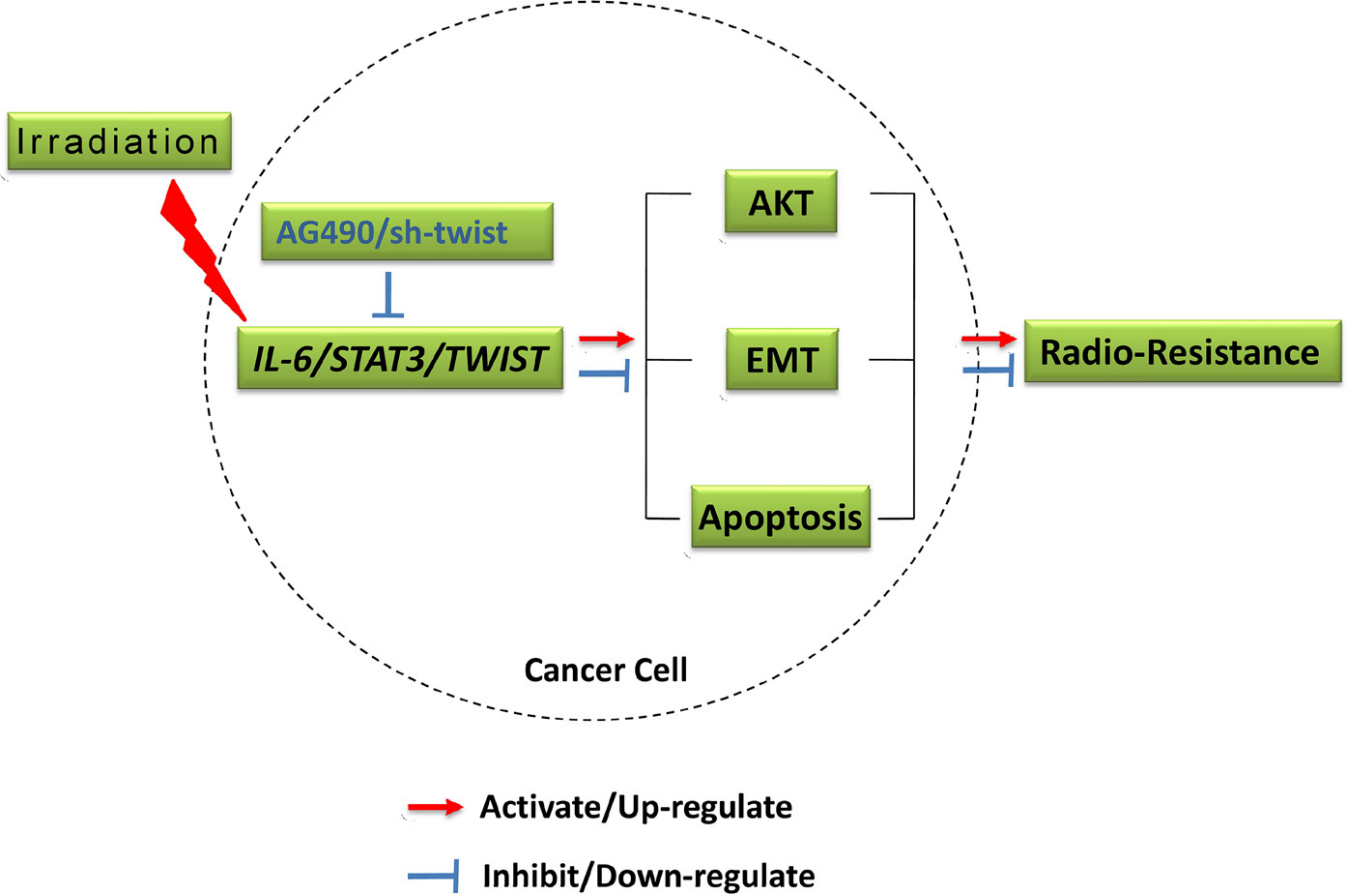
Outline diagram summarizing the mechanisms

Collectively, this study elucidated the role of IL-6/STAT3/TWIST pathway in IR triggered EMT and radioresistance in ESC, and more importantly highlighted the possibility of this pathway as potential therapeutic targets. Inhibition of IR triggered STAT3 activation and TWIST expression, which evokes the cascades leading to EMT (summarized in Figure 5), could be a useful strategy to reverse acquisition radioresistance in ESC.

## MATERIALS AND METHODS

### Cell culture, plasmids and reagents

The human ESC cell lines Eca109 were purchased from the Shanghai Cell Bank (Chinese Academy of Sciences) and cultured in RPMI1640 medium (Gibco, Waltham, USA) supplemented with 10% fetal bovine serum (Gibco, Waltham, USA) at 37°C in a 5% CO_2_ atmosphere.

The radioresistant cell line Eca109R was established by exposing Eca109 cells to 25 doses of 2Gy irradiation (total dose of 50 Gy), as previously reported [22]. To establish twist knockdown cells, four shRNA targeting twist mRNA and one negative control were designed (Supplementary Table 1), synthesized, and cloned separately into pYr-3.1 vector previously digested with BbsI and BamHI endonucleases. The pYr-3.1-shRNA plasmids were transfected into Eca109 or Eca109R cells with Lipofectamine 3000 according to the manufacturer's instructions (Thermo Fisher Scientific, NY). The cells were cultured in G418 (800 μg/ml) to screen for stable transfectants, and the selected twist-silenced or control cells were cultured long-term in medium supplemented with 400 μg/ml G418.

### IR treatment of cells and xenografts

Cells were cultured in flasks and irradiated with 6 MV X-rays at a dose rate of 200 cGy/min. The source-skin distance was set at 100 cm to the bottom of the flask and the exposure field was 20 cm × 20 cm. For experiments investigating pathway inhibition, the JAK2 inhibitor AG490 was added to the cells during IR treatment.

All animal experiments were performed in accordance with the Guidelines of the Chongqing Medical University Animal Care and Use Committee. Each cell line (2 × 10^6^ in PBS) was injected into the right upper flank of 5-week-old female BALB/c nude mice. Tumors were exposed to IR when they reached 50 mm^3^ in volume. Briefly, the source-skin distance was set at 100 cm, and the tumors were exposed to 6 MV X-rays at a dose rate of 200 cGy/min and 2 Gy/day for 5 consecutive days (total dose 10 Gy).

### Colony-forming assay

To evaluate radiosensitivity, the cells were seeded into six-well plates and incubated at 37°C overnight. The next day, the cells were irradiated with a single dose of 0, 2, 4, 6, 8, or 10 Gy (6 MV X-rays, 200 cGy/min, source-skin distance:100 cm) and then incubated in a 5% CO_2_ incubator at 37°C for 14 days. Finally, the colonies were fixed with methanol, stained with Giemsa, and counted under a microscope (a colony was defined as > 50 cells). All experiments were performed in triplicate. The plating efficiency (PE) was calculated as (colony number/cell number seeded) × 100%, and the surviving fraction was calculated as (PE of irradiated cells/PE of control cells) × 100%.

### Quantitative real-time PCR

Quantitative real-time PCR (Q-PCR) was performed to measure E-cadherin, N-cadherin, vimentin, IL-6, twist, and stat3 mRNA levels. Total RNA was isolated from cells using TRIzol reagent (Takara, Dalian, China) according to the manufacturer's instructions. Glyceraldehyde 3-phosphate dehydrogenase (GAPDH) gene expression served as an internal control. Reactions were performed using a MyiQ thermal cycler (Bio-Rad, Hercules, CA). The Ct value of each gene was determined after normalization to GAPDH, and ^ΔΔ^Ct was calculated relative to the designated reference sample. The fold-change in gene expression was calculated from 2^-ΔΔCt^. PCR primer sequences are listed in Supplementary Table 2.

### Western blotting blot analysis

Cells were harvested, washed three times with PBS, and then lysed by using RIPA buffer. Protein concentrations were determined using BCA Protein Assay Reagent (Pierce Chemical, Rockford, IL). Equal amounts of total protein (50 μg per lysate) were separated by 10% sodium dodecylsulfate-polyacrylamide gel electrophoresis and then transferred onto nitrocellulose membranes at 250 V for 2 h. Membranes were blocked for 1 h with 5% (w/v) nonfat milk powder in Tris-buffered saline/0.1% Tween 20 and then incubated with primary antibodies: mouse anti-E-cadherin, N-cadherin, vimentin, TWIST STAT3, p-STAT3(705), BAX, AKT, p-AKT, BCL-2, cleaved caspase 3, and β-actin (Cell Signaling Technology, Beverly, MA) at 4°C overnight. The membranes were washed and incubated with a Dylight 800-conjugated goat anti-mouse secondary antibody (EarthOx, San Francisco, CA). Membranes were washed again, and the immunoblotted proteins were detected using an Odyssey Western Blotting Detection System (Gene, Hong Kong, China).

### Immunofluorescence assay

Immunofluorescence assays (IFA) were performed on cells grown on LabTek chamber slides or on frozen sections of tumor tissues. Cells or sections were fixed with 2% (v/v) paraformaldehyde in PBS for 15 min on ice and then blocked for 30 min with 2% BSA plus 1% normal goat serum. The cells and sections were incubated with monoclonal antibodies to human E-cadherin, N-cadherin, or vimentin at 4°C overnight, and then incubated with Dylight549- or FITC-conjugated secondary antibodies at room temperature for 1 h. Nuclei were stained with 4′,6-diamidino-2-phenylindole (DAPI). Finally, cells were imaged using an Olympus ix73 fluorescence microscope.

### Tumor immunohistochemistry

Immunohistochemistry (IHC) was performed on formalin-fixed, paraffin-embedded tumor tissues from ESC patients or xenografted mice. Multiple tissue sections (5-μm thick) were cut from each tumor, deparaffinizedin xylene, rehydrated in alcohol, and washed twice. Sections were blocked for 30 min with 4% (w/v) nonfat milk powder in PBS and then incubated overnight at 4°C with mouse anti-human monoclonal antibodies to E-cadherin, N-cadherin, vimentin, IL-6, p-STAT3 and TWIST. The sections were washed and incubated with a horseradish peroxidase-conjugated goat anti-mouse IgG secondary antibody. Antibody binding was detected with a Vectasta in Elite ABC kit (Vector Laboratories, Burlingame, CA) following the manufacturer's procedure. The slides were examined under an Olympus microscope.

### Scratch assay

Cells were exposed to IR and then passaged in 60-mm dishes to confluence. The cell monolayer was scratched in a straight line with a 10-μl pipet tip. Debris was removed and the scratch edge was smoothed by washing the monolayer once with 1 ml PBS. RPMI1640 medium supplemented with 1.5% FBS was added and the cells were incubated at 37°C. Images were acquired at 0 h and again at 24 h using the markings on the culture dish as a reference. Migration was calculated with ImagePro Plus software: distance migrated (mm) = (scratch width at 0 h [mm] − scratch width at 24 h [mm]).

### Transwell invasion assay

Cell invasion was measured using a chemotaxis chamber (Corning Life Sciences, Lowell, MA). Briefly, after exposure to IR, cells were added to the upper chamber (1 × 10^5^ in 200 μl of culture medium) and the lower chamber was filled with 600 μl of culture medium. A polycarbonate membrane was placed between the two chambers. The cells were incubated in a humidified 5% CO_2_ incubator at 37°C for 24 h to allow invasion into the lower chamber. The non-migrating cells on the upper surface were washed away with ice-cold PBS, and the invaded cells on the lower surface of the membrane were stained with Giemsa and counted under a light microscope.

### IL-6 ELISA

IL-6 production by cells exposed to 0, 4, or 8 Gy of IR was measured using an ELISA kit (Abcam, Cambridge, MA). Briefly, the culture supernatants or IL-6 standard preparations (final concentrations: 1000, 500, 250, 125, 62.5, 31.2, and 15.6 pg/ml) were added to the ELISA plate wells and incubated. Biotinylated anti-IL-6 antibody, horseradish peroxidase-coupled streptavidin solution, 3,3′,5,5′-tetramethylbenzidine (TMB), and Stop Solution were added to the wells sequentially. The absorbance (optical density [OD]) at 450 nm was measured immediately in microplate reader (MK3, Thermo Fisher Scientific, Shanghai, China).

### Flow cytometric apoptosis assay

Cells were harvested by trypsinization and pipetted to give a single cell suspension. According to the instruction of apoptosis assay (Applygen, Beijing, China), aliquots of 1 × 10^5^ cells were resuspended in 500 μl of 1×Binding Buffer, and 5 μl of annexin V-FITC and 5 μl of propidium iodide (50 μg/ml) were added. Cells were incubated at room temperature for 5 min in the dark. Annexin V-FITC binding to cells was analyzed by flow cytometry (excitation 488 nm, emission 530 nm).

### Viability assay

Cells were resuspended at 5 × 10^4^ cells/ml, seeded in 96-well plates at 100 μl/well, and incubated for 24 h. The cells were then exposed to doses of 0, 4, or 8 Gy. For the assay, 20 μl of a 5 mg/ml solution of MTT was added to each well and the cells were incubated for 3 h in the dark. The supernatants were removed and 150 μl DMSO was added to each well. After 15 min incubation, the absorbance at 492 nm was measured with a microplate reader. Cell viability was calculated as ([OD value of IR treatment sample−OD value of IR treatment blank well]/[OD value of control sample−OD value of control blank well]) × 100%.

### Statistical analysis

Quantitative data are expressed as the mean ± standard deviation. Mean values were compared using one-way analysis of variance (ANOVA) and Student's *t*-test. A *P* value < 0.05 was considered statistically significant. All experiments were repeated 3 times

## SUPPLEMENTARY MATERIALS FIGURES AND TABLES


