# Supplementary material for: IL-6/STAT3/TWIST inhibition reverses ionizing radiation-induced EMT and radioresistance in esophageal squamous carcinoma
Source: Oncotarget. 2017 Jan 4;8(7):11228–38. doi: 10.18632/oncotarget.14495 (PMC5355260; doi:10.18632/oncotarget.14495)
Supplement: Supplementary file 1 [file oncotarget-08-11228-s001.pdf]

IL-6/STAT3/TWIST inhibition reverses ionizing radiation-induced EMT and radioresistance in esophageal squamous carcinoma

Supplementary Materials

Supplementary Table 1: The sequences of sh-Twist

No.	sh-Twist Sequence* (Sense strand)	Target Sequence
Sh-Twist-775	5′-GAAGCTGAGCAAGATTCAGACCTTCAA GAGAGGTCTGAATCTTGCTCAGCTTTTTTTTG-3′	5′-AAGCTGAGCAAGATTCAGACC-3′
Sh-Twist--812	5′-GGTACATCGACTTCCTCTACCTTCAAGAGA GGTAGAGGAAGTCGATGTACCTTTTTTG-3′	5′-GGTACATCGACTTCCTCTACC-3′
Sh-Twist -864	5′-GATGGCAAGCTGCAGCTATGTTTCAAGAGAAC ATAGCTGCAGCTTGCCATCTTTTTTG-3′	5′-GATGGCAAGCTGCAGCTATGT-3′
Sh-Twist -996	5′-GACCTAGATGTCATTGTTTCCTTCAAGAGAGG AAACAATGACATCTAGGTCTTTTTTG-3′	5′-GACCTAGATGTCATTGTTTCC-3′

*shRNA was designed according to twist mRNA uploaded in GenBank (NM_000474). 5′-TTCAAGAGA-3′ (in bold) was designed as loop structure.

Supplementary Table 2: The sequence of primers

Gene	The sequence of primers
E-Cadherin	Forward primer(5′-3′): TTGCTACTGGAACAGGGACACT<br>Reverse primer(5′-3′): GGAGATGTATTGGGAGGAAGGTC
N-Cadherin	Forward primer(5′-3′): CATCCTGCTTATCCTTGTGCTG<br>Reverse primer(5′-3′): CTGGTCTTCTTCTCCTCCACCTT
Vimentin	Forward primer(5′-3′): AAGGAGGAAATGGCTCGTCAC<br>Reverse primer(5′-3′): CTCAGGTTCAGGGAGGAAAAGT
IL-6	Forward primer(5′-3′):GGGAACGAAAGAGAAGCTCT<br>Reverse primer(5′-3′):ACCAGAAGAAGGAATGCCCA
Stat3	Forward primer(5′-3′): GAGGCCAACATACATGCCTTC<br>Reverse primer(5′-3′): GCTCCCGCTCCTTACTGATAAAG
Twist	Forward primer(5′-3′): AGCAAGATTCAGACCCTCAAGC<br>Forward primer(5′-3′): AGCAAGATTCAGACCCTCAAGC
GAPDH	Forward primer(5′-3′): TGACTTCAACAGCGACACCCA<br>Reverse primer(5′-3′): CACCCTGTTGCTGTAGCCAAA

Supplementary Table 3: Concentration of IL-6 (pg/ml) detected by ELISA

dose	24 h	48 h
0 Gy	209.19 ± 8.24	209.19 ± 8.24
4 Gy	305.29 ± 18.32*	344.14 ± 25.66*
8 Gy	266.42 ± 11.18*	285.22 ± 8.48*

The data represent the mean ± SEM of three independent experiments. * means significant difference $P < 0.05$ compared with the value of cells under 0 Gy IR.

Supplementary Table 4: The radiobiology parameters of cells detected by colony-forming assay

Group	SF2	D0 (Gy)	Dq (Gy)	N	SERD0	SERDq
Eca109	0.74 ± 0.01	3.14 ± 0.17	1.87 ± 0.14	1.81 ± 0.38	1	1
Eca109+4 Gy	0.81 ± 0.02*	3.48 ± 0.22*	2.49 ± 0.25*	2.04 ± 0.69*	0.89 ± 0.01*	0.75 ± 0.03*
Eca109R	0.89 ± 0.01*	3.92 ± 0.35*	3.59 ± 0.27**	2.50 ± 0.82**	0.80 ± 0.10**	0.52 ± 0.06**

The data represent the mean ± SEM of three independent experiments. * and ** on behalf of significant difference $P < 0.05$ and $P < 0.01$ respectively compared with Eca109 group.

Supplementary Table 5: Sensitizing irradiation detected by colony-forming assay

Group	SF2	D0 (Gy)	Dq (Gy)	N	SERD0	SERDq
Eca109R/sh-NC	0.89 ± 0.01	3.92 ± 0.35	3.59 ± 0.27	2.50 ± 0.82	1	1
Eca109R/sh-Twist	0.71 ± 0.03*	3.20 ± 0.24*	1.53 ± 0.31**	1.61 ± 0.47**	1.22 ± 0.17*	2.36 ± 0.16**

The data represent the mean ± SEM of three independent experiments. * and ** on behalf of significant difference $P < 0.05$ and $P < 0.01$ respectively compared with Eca109R/sh-NC group.

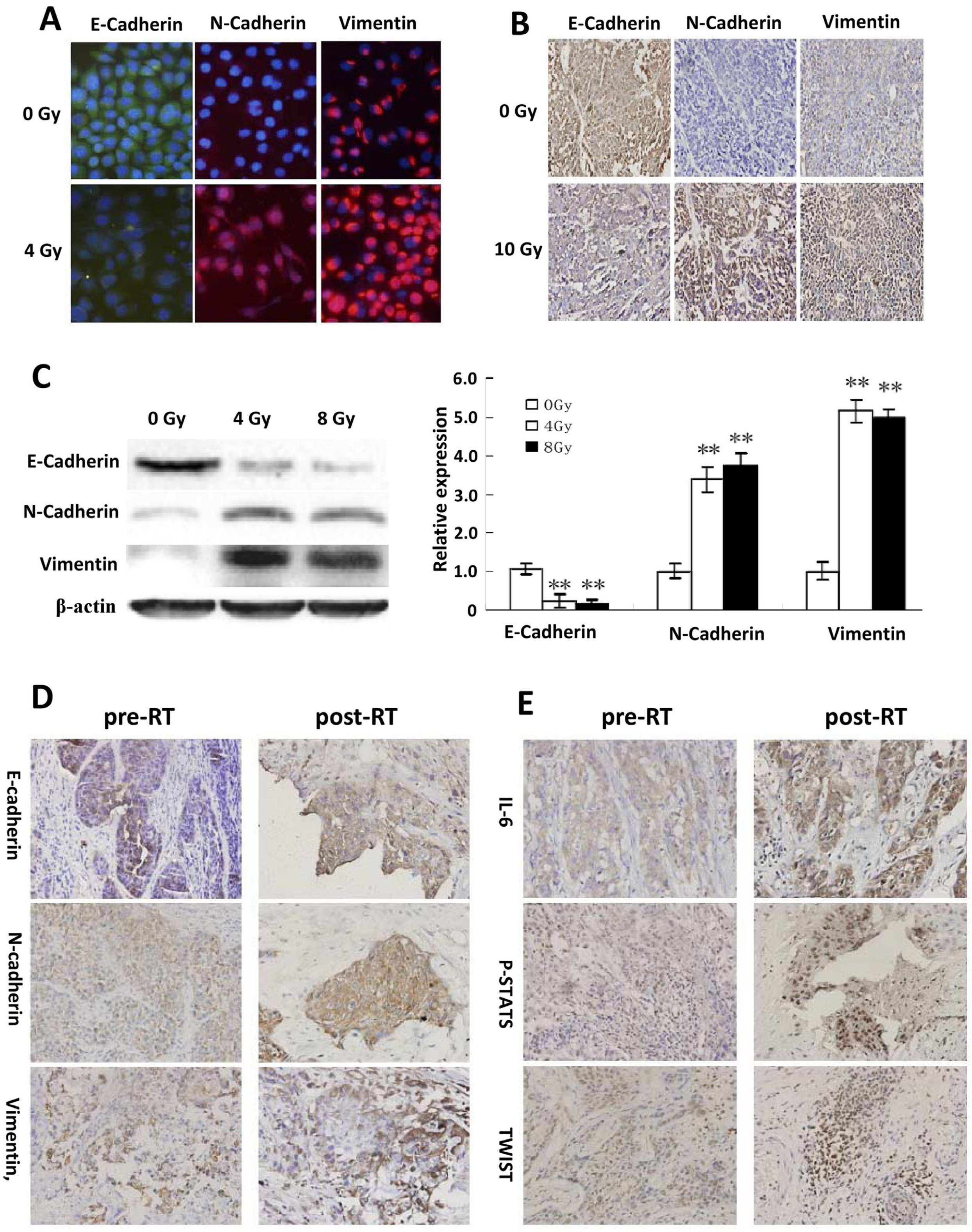

Supplementary Figure 1: (**A**) Immunofluorescence detection of the EMT biomarkers expression. (**B**) Immunohistochemistry detection of the EMT biomarkers expression in xenograft tumors. (**C**) Western-blotting detection of the EMT biomarkers expression (left) and their quantitative analysis (right). (**D**) Immunohistochemistry detection of the EMT biomarkers expression in ESC patients. (**E**) Immunohistochemistry detection of the IL-6/STAT3/TWIST pathway activation in ESC patients.

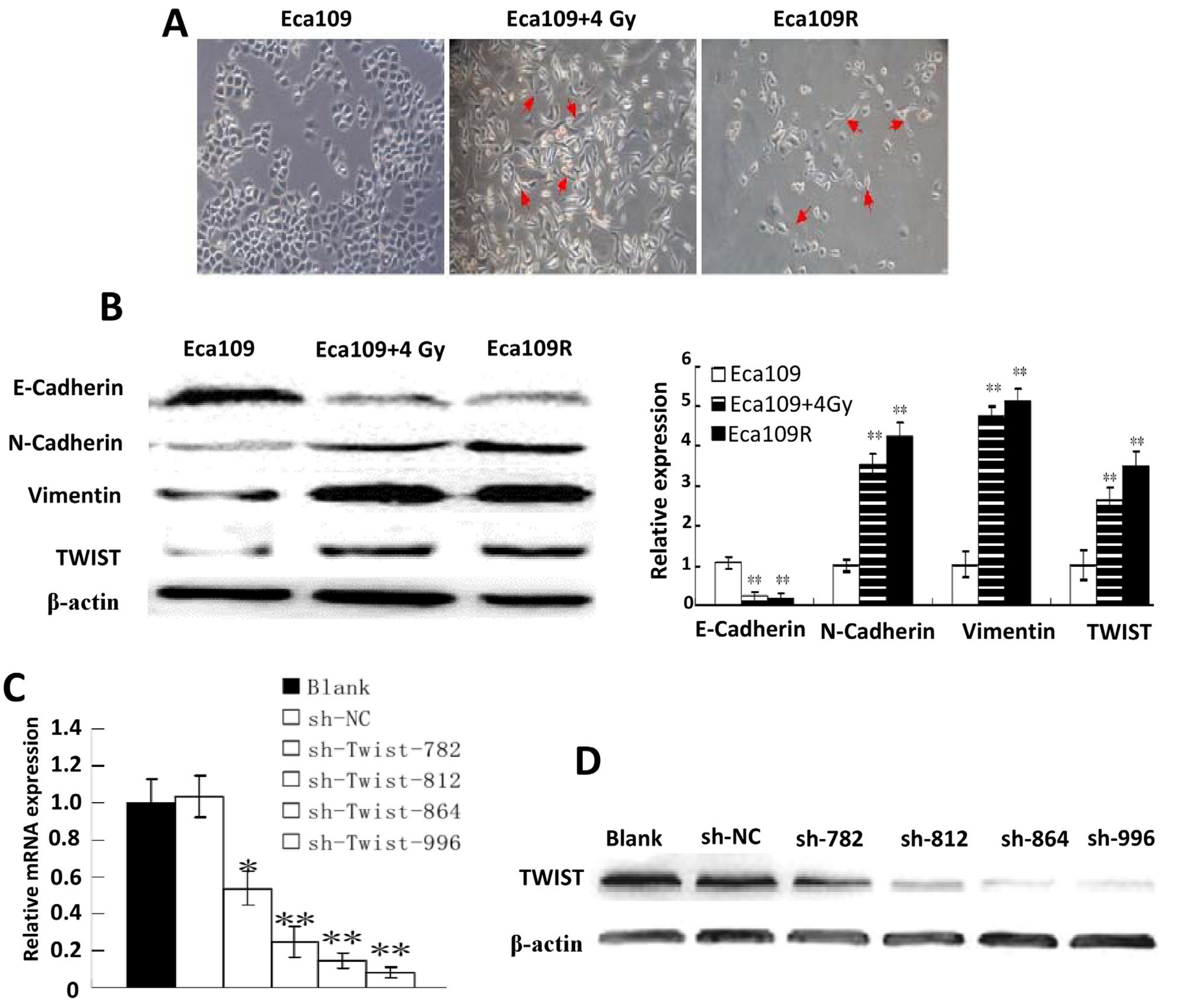

Supplementary Figure 2: (**A**) Representative morphological changes in Eca109R and Eca109 + 4 Gy cells; (**B**) Western-blotting detection of the EMT biomarkers expression in Eca109R and Eca109 + 4 Gy cells (left) and their quantitative analysis (right). (**C**) Quantitative PCR detection of the silenced twist mRNA induced by the four shRNA. (**D**) Western-blotting detection of TWIST expression to verify the efficacy of the four shRNA.

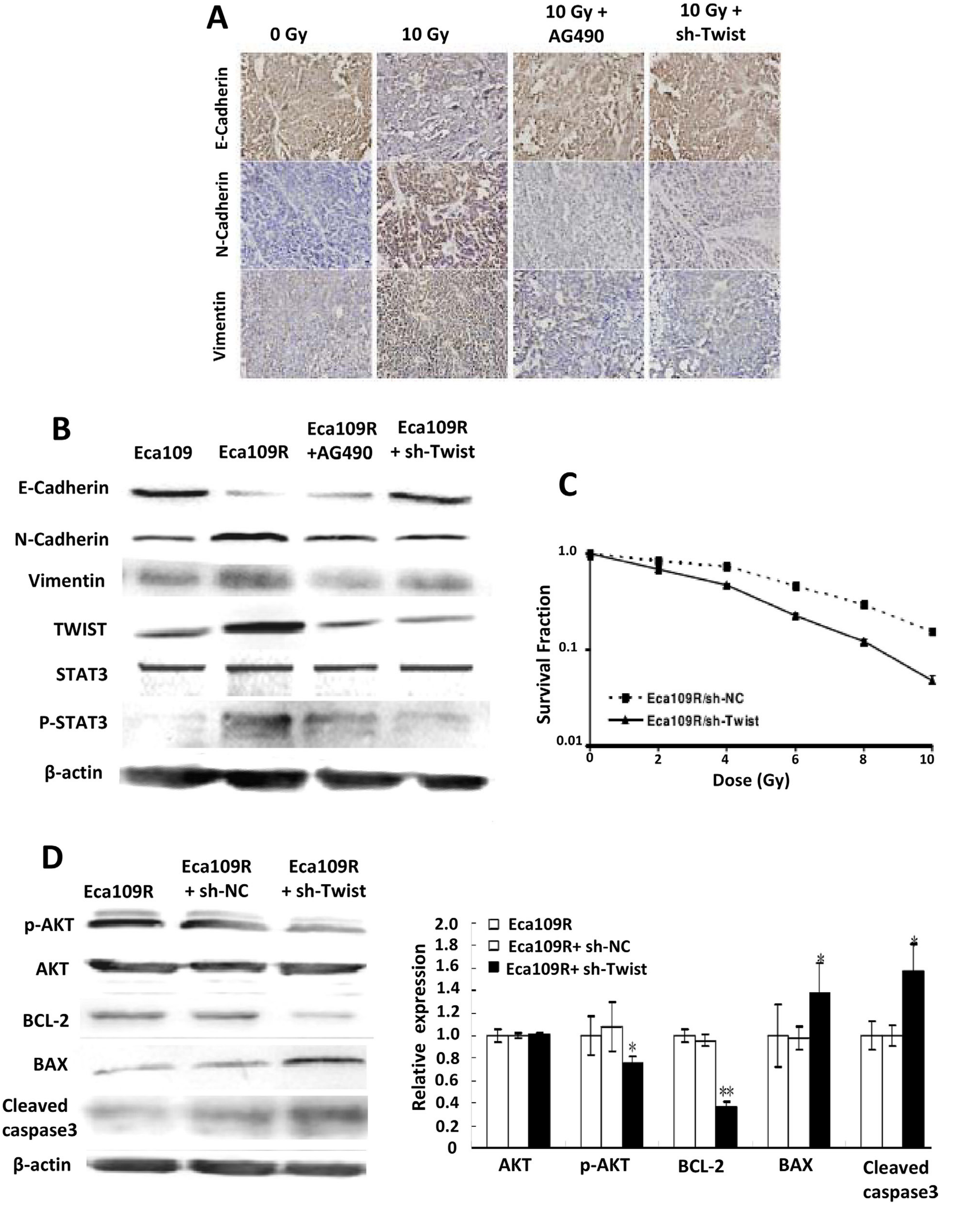

Supplementary Figure 3: (**A**) Immunofluorescence detection of the EMT biomarkers expression in xenografted tumors exposed to IR, AG490 or sh-Twist. (**B**) Western-blotting detection of the EMT biomarkers change and IL-6 pathway inhibition under AG490 or sh-Twist treatment. (**C**) Eca109R cells' revered radioresistance detected by colony-forming assay. (**D**) Western-blotting detection of apoptosis related proteins and AKT activation (left), and their quantitative analysis (right).
